# Supplementary material for: Detailed molecular and epigenetic characterization of the pig IPEC-J2 and chicken SL-29 cell lines
Source: iScience. 2023 Feb 20;26(3):106252. doi: 10.1016/j.isci.2023.106252 (PMC10018572; doi:10.1016/j.isci.2023.106252)
Supplement: Data S1. Complete homer output for identified motifs in Pig IPECJ-2, related to Table 2 — Homer motif analysis results for histone modifications H3K4me1, H3K4me3, H3K27ac, and enhancer elements of pig IPECJ2 cell line. P-values >1e-10 are possible false positives. Within each folder (e.g. peak_files_CTCF) are the html files showing the identified motifs when using homer (e.g. homerResults.html). [file mmc2.zip › S5/Pig_IPECJ_2/peak_files_H3K4me3/knownResults.html]

peak\_files\_H3K4me3 - Homer Known Motif Enrichment Results


# Homer Known Motif Enrichment Results (peak\_files\_H3K4me3)

Homer *de novo* Motif Results  
Gene Ontology Enrichment Results  
Known Motif Enrichment Results (txt file)  
Total Target Sequences = 22051, Total Background Sequences = 26455

|  |  |  |  |  |  |  |  |  |  |  |  |
| --- | --- | --- | --- | --- | --- | --- | --- | --- | --- | --- | --- |
| Rank | Motif | Name | P-value | log P-pvalue | q-value (Benjamini) | # Target Sequences with Motif | % of Targets Sequences with Motif | # Background Sequences with Motif | % of Background Sequences with Motif | Motif File | SVG |
| 1 | C T A G T C G A C T G A C G T A T A C G G A C T T C A G T C G A G T C A T G C A T A C G A G C T | IRF2(IRF)/Erythroblas-IRF2-ChIP-Seq(GSE36985)/Homer | 1e-246 | -5.681e+02 | 0.0000 | 871.0 | 3.95% | 261.9 | 0.99% | motif file (matrix) | svg |
| 2 | T C A G C T G A C G T A C G T A T A C G G C A T C T A G C T G A C G T A C G T A T A C G G A C T | IRF1(IRF)/PBMC-IRF1-ChIP-Seq(GSE43036)/Homer | 1e-159 | -3.672e+02 | 0.0000 | 924.0 | 4.19% | 397.8 | 1.50% | motif file (matrix) | svg |
| 3 | C T G A A T C G A G C T A G C T A C G T T A G C C T G A T A C G C G A T A C G T G A C T A G T C | ISRE(IRF)/ThioMac-LPS-Expression(GSE23622)/Homer | 1e-150 | -3.462e+02 | 0.0000 | 535.0 | 2.42% | 161.9 | 0.61% | motif file (matrix) | svg |
| 4 | C T G A T A C G G C A T A G C T A G C T A G T C T C G A A C T G C A G T A G C T A G C T G A T C | IRF3(IRF)/BMDM-Irf3-ChIP-Seq(GSE67343)/Homer | 1e-144 | -3.330e+02 | 0.0000 | 1398.0 | 6.33% | 786.4 | 2.97% | motif file (matrix) | svg |
| 5 | T C A G T C A G G C T A C G T A T A C G G A C T T C A G T C G A C T G A C G T A T A C G G A C T | IRF8(IRF)/BMDM-IRF8-ChIP-Seq(GSE77884)/Homer | 1e-142 | -3.290e+02 | 0.0000 | 1531.0 | 6.94% | 900.7 | 3.40% | motif file (matrix) | svg |
| 6 | T C G A T G A C G C A T A G C T C A G T G A T C G C T A G A T C G A C T A C G T G C A T A G T C | PRDM1(Zf)/Hela-PRDM1-ChIP-Seq(GSE31477)/Homer | 1e-89 | -2.060e+02 | 0.0000 | 1841.0 | 8.34% | 1350.9 | 5.11% | motif file (matrix) | svg |
| 7 | C T A G C T A G C G T A C G T A T A C G C G A T C T A G C T G A C T G A C G T A T A C G G A C T | PU.1:IRF8(ETS:IRF)/pDC-Irf8-ChIP-Seq(GSE66899)/Homer | 1e-84 | -1.935e+02 | 0.0000 | 794.0 | 3.60% | 440.1 | 1.66% | motif file (matrix) | svg |
| 8 | G C T A T A G C A G C T A T C G G T C A C G T A G C T A A T G C G A T C C T G A | IRF4(IRF)/GM12878-IRF4-ChIP-Seq(GSE32465)/Homer | 1e-47 | -1.104e+02 | 0.0000 | 1309.0 | 5.93% | 1028.4 | 3.89% | motif file (matrix) | svg |
| 9 | C T A G C T A G A G T C T C A G A C T G A C G T A C G T C T G A | MYB(HTH)/ERMYB-Myb-ChIPSeq(GSE22095)/Homer | 1e-35 | -8.194e+01 | 0.0000 | 5067.0 | 22.95% | 5171.0 | 19.54% | motif file (matrix) | svg |
| 10 | A G T C G C A T C G T A C G T A G T A C A C G T A C T G G A T C G A T C T C G A | BMYB(HTH)/Hela-BMYB-ChIP-Seq(GSE27030)/Homer | 1e-34 | -7.909e+01 | 0.0000 | 4126.0 | 18.69% | 4130.6 | 15.61% | motif file (matrix) | svg |
| 11 | T G A C C T A G T C A G G T C A C G T A T C A G C G A T T C A G T C G A T G C A C T G A T A G C | PU.1-IRF(ETS:IRF)/Bcell-PU.1-ChIP-Seq(GSE21512)/Homer | 1e-33 | -7.642e+01 | 0.0000 | 4111.0 | 18.62% | 4128.0 | 15.60% | motif file (matrix) | svg |
| 12 | A C T G C A T G T C G A A C G T A C T G C G T A A T C G A C G T G T A C C G T A G A C T A G T C | Fos(bZIP)/TSC-Fos-ChIP-Seq(GSE110950)/Homer | 1e-30 | -7.090e+01 | 0.0000 | 1257.0 | 5.69% | 1074.8 | 4.06% | motif file (matrix) | svg |
| 13 | T A G C T A G C G C A T C A T G A C T G G C T A C G T A A C G T A C T G G A T C | TEAD4(TEA)/Tropoblast-Tead4-ChIP-Seq(GSE37350)/Homer | 1e-29 | -6.755e+01 | 0.0000 | 2081.0 | 9.43% | 1946.0 | 7.35% | motif file (matrix) | svg |
| 14 | C T A G T C G A G C A T C A T G G C T A T A G C C G A T G T A C C T G A A G C T | JunB(bZIP)/DendriticCells-Junb-ChIP-Seq(GSE36099)/Homer | 1e-27 | -6.446e+01 | 0.0000 | 1171.0 | 5.30% | 1005.2 | 3.80% | motif file (matrix) | svg |
| 15 | T C G A T C G A T A C G G A T C G T C A G T A C C G A T A G C T G T C A T G C A | Nkx3.1(Homeobox)/LNCaP-Nkx3.1-ChIP-Seq(GSE28264)/Homer | 1e-27 | -6.266e+01 | 0.0000 | 5320.0 | 24.10% | 5572.2 | 21.06% | motif file (matrix) | svg |
| 16 | C T A G T C G A A C G T A C T G C G T A A T G C A C G T G T A C C G T A A G C T G A T C G T A C | Atf3(bZIP)/GBM-ATF3-ChIP-Seq(GSE33912)/Homer | 1e-27 | -6.257e+01 | 0.0000 | 1394.0 | 6.32% | 1240.3 | 4.69% | motif file (matrix) | svg |
| 17 | A G C T C G A T T A C G A T C G A T C G C A G T A G T C A G T C A C T G T A G C | HINFP(Zf)/K562-HINFP.eGFP-ChIP-Seq(Encode)/Homer | 1e-25 | -5.793e+01 | 0.0000 | 3585.0 | 16.24% | 3640.3 | 13.76% | motif file (matrix) | svg |
| 18 | A C T G C T A G T C G A C G A T C A T G G C T A A T C G C G A T G T A C G C T A A G C T G T A C | Fra1(bZIP)/BT549-Fra1-ChIP-Seq(GSE46166)/Homer | 1e-24 | -5.599e+01 | 0.0000 | 1185.0 | 5.37% | 1045.8 | 3.95% | motif file (matrix) | svg |
| 19 | C A G T T G C A A C G T A C T G C G T A A T C G C G A T T G A C C G T A A C G T | BATF(bZIP)/Th17-BATF-ChIP-Seq(GSE39756)/Homer | 1e-23 | -5.407e+01 | 0.0000 | 1331.0 | 6.03% | 1203.3 | 4.55% | motif file (matrix) | svg |
| 20 | T G C A A T G C A C G T A C G T A C G T A T G C C T A G A C G T A C G T A G C T G A T C A G C T | T1ISRE(IRF)/ThioMac-Ifnb-Expression/Homer | 1e-23 | -5.299e+01 | 0.0000 | 83.0 | 0.38% | 26.8 | 0.10% | motif file (matrix) | svg |
| 21 | C G A T C T G A C G T A C A G T A G T C G A T C G A T C A C T G | Pitx1(Homeobox)/Chicken-Pitx1-ChIP-Seq(GSE38910)/Homer | 1e-22 | -5.154e+01 | 0.0000 | 8950.0 | 40.55% | 9876.3 | 37.33% | motif file (matrix) | svg |
| 22 | A T C G G A T C G C A T C G T A G T C A A C G T A T G C A G T C | CRX(Homeobox)/Retina-Crx-ChIP-Seq(GSE20012)/Homer | 1e-21 | -5.059e+01 | 0.0000 | 4794.0 | 21.72% | 5053.0 | 19.10% | motif file (matrix) | svg |
| 23 | G A C T C T A G C T A G A G T C T G C A A C T G A C G T A C G T C T A G T C A G | AMYB(HTH)/Testes-AMYB-ChIP-Seq(GSE44588)/Homer | 1e-20 | -4.796e+01 | 0.0000 | 4036.0 | 18.28% | 4209.6 | 15.91% | motif file (matrix) | svg |
| 24 | C A T G C T A G T C G A A C G T A C T G C G T A T A G C C G A T T G A C C G T A A G C T G A T C | Fra2(bZIP)/Striatum-Fra2-ChIP-Seq(GSE43429)/Homer | 1e-19 | -4.578e+01 | 0.0000 | 1059.0 | 4.80% | 948.9 | 3.59% | motif file (matrix) | svg |
| 25 | C T A G T C G A C G A T A C T G C G T A T A C G A G C T T G A C G C T A A C G T G A T C T A G C | Fosl2(bZIP)/3T3L1-Fosl2-ChIP-Seq(GSE56872)/Homer | 1e-19 | -4.432e+01 | 0.0000 | 761.0 | 3.45% | 647.8 | 2.45% | motif file (matrix) | svg |
| 26 | T C G A A C G T C A T G G C T A T A G C C G A T G T A C G C T A A C G T A T G C | AP-1(bZIP)/ThioMac-PU.1-ChIP-Seq(GSE21512)/Homer | 1e-18 | -4.322e+01 | 0.0000 | 1544.0 | 6.99% | 1471.7 | 5.56% | motif file (matrix) | svg |
| 27 | A T G C C T G A A T C G T A C G A G T C C G A T T C A G C G A T C T A G A G C T G T C A G T C A C G T A A G T C C G T A T A C G C T G A | Fox:Ebox(Forkhead,bHLH)/Panc1-Foxa2-ChIP-Seq(GSE47459)/Homer | 1e-18 | -4.280e+01 | 0.0000 | 1863.0 | 8.44% | 1818.8 | 6.87% | motif file (matrix) | svg |
| 28 | T G A C A G T C C T G A T G A C C G T A A C G T A C G T A G T C A G T C C G T A | TEAD1(TEAD)/HepG2-TEAD1-ChIP-Seq(Encode)/Homer | 1e-18 | -4.272e+01 | 0.0000 | 2257.0 | 10.22% | 2250.4 | 8.50% | motif file (matrix) | svg |
| 29 | G A T C G T A C C G A T A C T G A C T G C G T A C G T A A C G T A C T G G A T C | TEAD(TEA)/Fibroblast-PU.1-ChIP-Seq(Unpublished)/Homer | 1e-17 | -4.095e+01 | 0.0000 | 1424.0 | 6.45% | 1352.5 | 5.11% | motif file (matrix) | svg |
| 30 | T A C G A T G C G C T A A C T G C G T A A C G T C G T A C T G A T A C G T C G A | Gata4(Zf)/Heart-Gata4-ChIP-Seq(GSE35151)/Homer | 1e-17 | -4.062e+01 | 0.0000 | 1973.0 | 8.94% | 1949.4 | 7.37% | motif file (matrix) | svg |
| 31 | A G C T G A C T C T A G C G T A C A T G C G A T C T A G A T C G G A C T C A G T | Bapx1(Homeobox)/VertebralCol-Bapx1-ChIP-Seq(GSE36672)/Homer | 1e-17 | -4.018e+01 | 0.0000 | 4743.0 | 21.49% | 5072.8 | 19.17% | motif file (matrix) | svg |
| 32 | C T A G T C G A A C G T A C T G C G T A T A G C C G A T G T A C C G T A A G C T G A T C G T A C | Jun-AP1(bZIP)/K562-cJun-ChIP-Seq(GSE31477)/Homer | 1e-17 | -3.948e+01 | 0.0000 | 553.0 | 2.51% | 452.5 | 1.71% | motif file (matrix) | svg |
| 33 | A T G C G A T C C G A T C T A G A C T G G C T A C G T A A G C T A C T G A G C T | TEAD2(TEA)/Py2T-Tead2-ChIP-Seq(GSE55709)/Homer | 1e-15 | -3.580e+01 | 0.0000 | 1182.0 | 5.35% | 1115.2 | 4.21% | motif file (matrix) | svg |
| 34 | T A G C G C T A A C T G C G T A A C G T C G T A C G T A T A C G T C A G T C G A | Gata1(Zf)/K562-GATA1-ChIP-Seq(GSE18829)/Homer | 1e-15 | -3.510e+01 | 0.0000 | 1168.0 | 5.29% | 1103.9 | 4.17% | motif file (matrix) | svg |
| 35 | G C T A A T C G G C T A G A C T G C T A T C G A T A G C T C G A | GATA3(Zf)/iTreg-Gata3-ChIP-Seq(GSE20898)/Homer | 1e-15 | -3.477e+01 | 0.0000 | 2715.0 | 12.30% | 2807.1 | 10.61% | motif file (matrix) | svg |
| 36 | A G C T A T G C G A C T G C A T C G T A A G C T G T A C C G A T A T C G A G T C | Gata6(Zf)/HUG1N-GATA6-ChIP-Seq(GSE51936)/Homer | 1e-15 | -3.471e+01 | 0.0000 | 1756.0 | 7.96% | 1743.0 | 6.59% | motif file (matrix) | svg |
| 37 | A G C T A G T C A T G C A G C T A C G T C G T A A C G T A G T C C G A T A T G C | Gata2(Zf)/K562-GATA2-ChIP-Seq(GSE18829)/Homer | 1e-14 | -3.450e+01 | 0.0000 | 1298.0 | 5.88% | 1245.5 | 4.71% | motif file (matrix) | svg |
| 38 | A G T C C T G A A T C G A G C T A G C T G A C T A G T C G C T A A C G T C G A T G C A T C G A T A T C G C G T A T A G C G C A T A T G C C G T A | bZIP:IRF(bZIP,IRF)/Th17-BatF-ChIP-Seq(GSE39756)/Homer | 1e-14 | -3.400e+01 | 0.0000 | 1035.0 | 4.69% | 966.3 | 3.65% | motif file (matrix) | svg |
| 39 | A C G T C T A G A G C T A C G T A C G T C T G A A G T C G A C T A G C T C G T A | FOXM1(Forkhead)/MCF7-FOXM1-ChIP-Seq(GSE72977)/Homer | 1e-14 | -3.381e+01 | 0.0000 | 1955.0 | 8.86% | 1966.1 | 7.43% | motif file (matrix) | svg |
| 40 | T A C G T A C G G T A C A T C G T A C G T A C G G T C A C T G A C G T A G A C T | E2F4(E2F)/K562-E2F4-ChIP-Seq(GSE31477)/Homer | 1e-14 | -3.371e+01 | 0.0000 | 3609.0 | 16.35% | 3826.8 | 14.46% | motif file (matrix) | svg |
| 41 | C T A G C G T A G T C A C G T A A G T C G A T C A G C T C T A G C G T A A C G T G T C A G A T C | Six2(Homeobox)/NephronProgenitor-Six2-ChIP-Seq(GSE39837)/Homer | 1e-14 | -3.330e+01 | 0.0000 | 2194.0 | 9.94% | 2234.1 | 8.44% | motif file (matrix) | svg |
| 42 | A G T C G A C T C A G T G T A C A G T C A T C G T C A G A C T G G T C A C G T A | Stat3(Stat)/mES-Stat3-ChIP-Seq(GSE11431)/Homer | 1e-13 | -2.995e+01 | 0.0000 | 1616.0 | 7.32% | 1614.3 | 6.10% | motif file (matrix) | svg |
| 43 | A G T C A C G T A C T G A G C T A C G T A C G T G T C A A G T C | Foxo1(Forkhead)/RAW-Foxo1-ChIP-Seq(Fan\_et\_al.)/Homer | 1e-12 | -2.988e+01 | 0.0000 | 4423.0 | 20.04% | 4791.2 | 18.11% | motif file (matrix) | svg |
| 44 | A G C T C T A G T G A C C G T A A C G T C G A T A G T C A G T C C T G A C A T G | TEAD3(TEA)/HepG2-TEAD3-ChIP-Seq(Encode)/Homer | 1e-12 | -2.857e+01 | 0.0000 | 2454.0 | 11.12% | 2556.0 | 9.66% | motif file (matrix) | svg |
| 45 | G A T C T C G A A G T C C G A T C G A T A G T C A T G C A C T G A T C G G A C T | Elk1(ETS)/Hela-Elk1-ChIP-Seq(GSE31477)/Homer | 1e-12 | -2.806e+01 | 0.0000 | 3027.0 | 13.71% | 3208.2 | 12.12% | motif file (matrix) | svg |
| 46 | C T G A T A G C T G A C T C A G C T A G G T C A C G T A T C A G A G C T T C A G | ETV4(ETS)/HepG2-ETV4-ChIP-Seq(ENCODE)/Homer | 1e-12 | -2.806e+01 | 0.0000 | 4898.0 | 22.19% | 5357.9 | 20.25% | motif file (matrix) | svg |
| 47 | A T G C G A C T A C G T C T A G A C G T A C G T A C G T C T G A G A T C G C T A A G C T C G T A | Foxa2(Forkhead)/Liver-Foxa2-ChIP-Seq(GSE25694)/Homer | 1e-11 | -2.716e+01 | 0.0000 | 1520.0 | 6.89% | 1524.0 | 5.76% | motif file (matrix) | svg |
| 48 | G A T C C T G A A G T C C G A T C G A T G A T C A G T C A C T G A T C G A G C T | Elk4(ETS)/Hela-Elk4-ChIP-Seq(GSE31477)/Homer | 1e-11 | -2.620e+01 | 0.0000 | 3099.0 | 14.04% | 3306.0 | 12.49% | motif file (matrix) | svg |
| 49 | C T G A C T G A C T G A A T G C G A T C C A T G A C T G G A C T G A C T G C A T C G T A C G T A A G T C G T A C C T G A A T C G G C A T G A C T G A C T A G C T | GRHL2(CP2)/HBE-GRHL2-ChIP-Seq(GSE46194)/Homer | 1e-11 | -2.551e+01 | 0.0000 | 749.0 | 3.39% | 697.8 | 2.64% | motif file (matrix) | svg |
| 50 | G C A T A T C G C A T G G T A C G C T A A G T C T C A G T G A C G T C A T G C A | Arnt:Ahr(bHLH)/MCF7-Arnt-ChIP-Seq(Lo\_et\_al.)/Homer | 1e-10 | -2.466e+01 | 0.0000 | 2262.0 | 10.25% | 2368.8 | 8.95% | motif file (matrix) | svg |
| 51 | C G T A A C T G C G T A A C G T C G T A C T G A T C A G T G C A A G C T T G A C | TRPS1(Zf)/MCF7-TRPS1-ChIP-Seq(GSE107013)/Homer | 1e-10 | -2.367e+01 | 0.0000 | 3546.0 | 16.06% | 3839.2 | 14.51% | motif file (matrix) | svg |
| 52 | A T G C G A C T A G C T A G C T A G T C G C T A C A G T C G A T G C T A A C G T A C T G G C T A T A G C G C A T T G A C | IRF:BATF(IRF:bZIP)/pDC-Irf8-ChIP-Seq(GSE66899)/Homer | 1e-10 | -2.360e+01 | 0.0000 | 300.0 | 1.36% | 242.8 | 0.92% | motif file (matrix) | svg |
| 53 | A G C T A G T C A G T C A C G T C T A G A C G T A C G T A C G T C G T A A G T C G A T C C G T A | FOXP1(Forkhead)/H9-FOXP1-ChIP-Seq(GSE31006)/Homer | 1e-9 | -2.111e+01 | 0.0000 | 896.0 | 4.06% | 874.0 | 3.30% | motif file (matrix) | svg |
| 54 | T C G A A G C T A C G T A C G T A G T C A G T C A C G T A T C G G A C T A T C G | EWS:ERG-fusion(ETS)/CADO\_ES1-EWS:ERG-ChIP-Seq(SRA014231)/Homer | 1e-9 | -2.108e+01 | 0.0000 | 1781.0 | 8.07% | 1853.8 | 7.01% | motif file (matrix) | svg |
| 55 | T C G A T A G C G T C A A C T G A C T G C G T A C G T A C T A G A G C T T C A G | ERG(ETS)/VCaP-ERG-ChIP-Seq(GSE14097)/Homer | 1e-8 | -2.066e+01 | 0.0000 | 4985.0 | 22.58% | 5537.8 | 20.93% | motif file (matrix) | svg |
| 56 | T C G A C T G A T A G C T G A C T C A G T C A G C G T A C G T A T C A G A G C T | ETV1(ETS)/GIST48-ETV1-ChIP-Seq(GSE22441)/Homer | 1e-8 | -2.062e+01 | 0.0000 | 4928.0 | 22.32% | 5471.5 | 20.68% | motif file (matrix) | svg |
| 57 | A C T G A C G T C A T G A T C G A T C G T G A C A C T G A T C G A T C G T G C A C T G A C G T A | E2F3(E2F)/MEF-E2F3-ChIP-Seq(GSE71376)/Homer | 1e-8 | -2.049e+01 | 0.0000 | 4943.0 | 22.39% | 5490.9 | 20.75% | motif file (matrix) | svg |
| 58 | T C G A C A T G C A T G T A C G G T A C T C G A A G T C C A G T C T G A C G T A A G T C G A C T | ZNF264(Zf)/HEK293-ZNF264.GFP-ChIP-Seq(GSE58341)/Homer | 1e-8 | -2.033e+01 | 0.0000 | 2309.0 | 10.46% | 2455.6 | 9.28% | motif file (matrix) | svg |
| 59 | A G T C C T G A A G T C C G A T C A G T G A T C A T G C A C T G A T C G G A C T | Fli1(ETS)/CD8-FLI-ChIP-Seq(GSE20898)/Homer | 1e-8 | -1.986e+01 | 0.0000 | 4564.0 | 20.68% | 5056.8 | 19.11% | motif file (matrix) | svg |
| 60 | T A G C T C A G C A T G G C A T A G C T C G A T A T G C C G T A C G T A G T C A | CHR(?)/Hela-CellCycle-Expression/Homer | 1e-8 | -1.965e+01 | 0.0000 | 1312.0 | 5.94% | 1339.8 | 5.06% | motif file (matrix) | svg |
| 61 | A G C T T G A C C G A T C G A T C T A G A C G T C A G T C A G T G C T A A G T C | FOXK1(Forkhead)/HEK293-FOXK1-ChIP-Seq(GSE51673)/Homer | 1e-8 | -1.948e+01 | 0.0000 | 1956.0 | 8.86% | 2062.3 | 7.79% | motif file (matrix) | svg |
| 62 | T G C A A G C T A C G T C T A G G A T C C T A G G A T C G T C A C T G A A G T C | CEBP(bZIP)/ThioMac-CEBPb-ChIP-Seq(GSE21512)/Homer | 1e-8 | -1.874e+01 | 0.0000 | 1051.0 | 4.76% | 1056.3 | 3.99% | motif file (matrix) | svg |
| 63 | G C T A T C G A C G T A C T A G A G C T G T C A G T C A C G T A A G T C C G T A | FOXA1(Forkhead)/LNCAP-FOXA1-ChIP-Seq(GSE27824)/Homer | 1e-7 | -1.825e+01 | 0.0000 | 2194.0 | 9.94% | 2342.2 | 8.85% | motif file (matrix) | svg |
| 64 | C G A T T A C G T G A C G A C T C A T G C G T A T A C G A C G T G T A C C T G A | Bach2(bZIP)/OCILy7-Bach2-ChIP-Seq(GSE44420)/Homer | 1e-7 | -1.802e+01 | 0.0000 | 447.0 | 2.03% | 408.3 | 1.54% | motif file (matrix) | svg |
| 65 | A T G C A G T C G A T C C G T A A C G T A C G T A C T G A C G T A G C T G A T C | Sox2(HMG)/mES-Sox2-ChIP-Seq(GSE11431)/Homer | 1e-7 | -1.795e+01 | 0.0000 | 1923.0 | 8.71% | 2037.8 | 7.70% | motif file (matrix) | svg |
| 66 | A G T C T A G C G A C T A C G T C T A G A C G T A C G T A C G T C T G A A G T C G C T A G A C T C G T A C T A G A C T G | Foxa3(Forkhead)/Liver-Foxa3-ChIP-Seq(GSE77670)/Homer | 1e-7 | -1.689e+01 | 0.0000 | 589.0 | 2.67% | 563.5 | 2.13% | motif file (matrix) | svg |
| 67 | T A G C C G A T T A C G A C T G A G T C A C T G A T C G A T C G C G T A C T G A | E2F1(E2F)/Hela-E2F1-ChIP-Seq(GSE22478)/Homer | 1e-7 | -1.681e+01 | 0.0000 | 2189.0 | 9.92% | 2349.5 | 8.88% | motif file (matrix) | svg |
| 68 | G A C T C G A T T C A G G A T C G A C T A G C T A G C T A G T C G A T C C G T A C T A G C T A G T C G A T C G A C T G A | Bcl6(Zf)/Liver-Bcl6-ChIP-Seq(GSE31578)/Homer | 1e-7 | -1.669e+01 | 0.0000 | 3344.0 | 15.15% | 3677.4 | 13.90% | motif file (matrix) | svg |
| 69 | T C G A T A G C T G C A A C T G A C T G C G T A C G T A C T A G G A C T T A C G | ETS1(ETS)/Jurkat-ETS1-ChIP-Seq(GSE17954)/Homer | 1e-6 | -1.593e+01 | 0.0000 | 3492.0 | 15.82% | 3857.1 | 14.58% | motif file (matrix) | svg |
| 70 | T A C G T A G C G C T A C G A T C T A G A C G T C A G T C A G T G C T A A G T C G T C A G C A T | FOXK2(Forkhead)/U2OS-FOXK2-ChIP-Seq(E-MTAB-2204)/Homer | 1e-6 | -1.552e+01 | 0.0000 | 1253.0 | 5.68% | 1301.2 | 4.92% | motif file (matrix) | svg |
| 71 | G C T A T C G A C G T A C T A G A G C T G T C A G T C A C G T A A G T C C G T A | FOXA1(Forkhead)/MCF7-FOXA1-ChIP-Seq(GSE26831)/Homer | 1e-6 | -1.546e+01 | 0.0000 | 1812.0 | 8.21% | 1932.6 | 7.30% | motif file (matrix) | svg |
| 72 | C G T A C G T A C G T A G C A T G C A T T A C G G T A C G A C T A C T G C G T A A T C G A C G T G T A C C G T A A G C T | Bach1(bZIP)/K562-Bach1-ChIP-Seq(GSE31477)/Homer | 1e-6 | -1.535e+01 | 0.0000 | 136.0 | 0.62% | 102.6 | 0.39% | motif file (matrix) | svg |
| 73 | C G T A T A G C T A G C T G C A A C T G C T A G C G T A C G T A T C A G G A C T | EHF(ETS)/LoVo-EHF-ChIP-Seq(GSE49402)/Homer | 1e-6 | -1.532e+01 | 0.0000 | 3765.0 | 17.06% | 4181.2 | 15.80% | motif file (matrix) | svg |
| 74 | A T G C T C G A A G T C A G C T A C G T G T A C A G T C G C T A C T A G C A T G G T C A C T G A T C A G A G T C | Stat3+il21(Stat)/CD4-Stat3-ChIP-Seq(GSE19198)/Homer | 1e-6 | -1.521e+01 | 0.0000 | 1869.0 | 8.47% | 1999.6 | 7.56% | motif file (matrix) | svg |
| 75 | G C T A G A C T G A C T T G C A C G T A A G T C C G T A T A G C G A T C G A C T | Eomes(T-box)/H9-Eomes-ChIP-Seq(GSE26097)/Homer | 1e-6 | -1.501e+01 | 0.0000 | 4276.0 | 19.37% | 4780.2 | 18.07% | motif file (matrix) | svg |
| 76 | C G A T C T A G A C G T G T C A C G T A C G T A A G T C C G T A | Foxo3(Forkhead)/U2OS-Foxo3-ChIP-Seq(E-MTAB-2701)/Homer | 1e-6 | -1.494e+01 | 0.0000 | 1357.0 | 6.15% | 1422.6 | 5.38% | motif file (matrix) | svg |
| 77 | A T G C C T G A G A C T A C G T A C G T G T A C G A T C C G A T C T A G C A T G C G T A C G T A C T G A G A C T | STAT1(Stat)/HelaS3-STAT1-ChIP-Seq(GSE12782)/Homer | 1e-6 | -1.493e+01 | 0.0000 | 682.0 | 3.09% | 673.3 | 2.54% | motif file (matrix) | svg |
| 78 | C T G A C T G A C A T G A T C G A G C T A T C G G A C T C A T G C T G A G T C A | Tbr1(T-box)/Cortex-Tbr1-ChIP-Seq(GSE71384)/Homer | 1e-6 | -1.394e+01 | 0.0000 | 2765.0 | 12.53% | 3039.5 | 11.49% | motif file (matrix) | svg |
| 79 | C T G A C T G A T A G C G A T C G C T A G T A C A C G T G A T C T G C A C G T A | Nkx2.5(Homeobox)/HL1-Nkx2.5.biotin-ChIP-Seq(GSE21529)/Homer | 1e-6 | -1.388e+01 | 0.0000 | 4968.0 | 22.51% | 5605.8 | 21.19% | motif file (matrix) | svg |
| 80 | T C G A T C G A T A G C G T A C T C A G T A C G C G T A C G T A T C A G A G C T | GABPA(ETS)/Jurkat-GABPa-ChIP-Seq(GSE17954)/Homer | 1e-5 | -1.358e+01 | 0.0000 | 3328.0 | 15.08% | 3695.5 | 13.97% | motif file (matrix) | svg |
| 81 | C T A G T A C G A G T C C G T A A G T C A C G T A G T C T C G A C G T A T A C G | Nkx2.1(Homeobox)/LungAC-Nkx2.1-ChIP-Seq(GSE43252)/Homer | 1e-5 | -1.301e+01 | 0.0000 | 6633.0 | 30.05% | 7579.1 | 28.64% | motif file (matrix) | svg |
| 82 | G C T A A G T C T A C G T G C A A T C G T C A G G C T A T C G A T C A G A G C T | ELF5(ETS)/T47D-ELF5-ChIP-Seq(GSE30407)/Homer | 1e-5 | -1.280e+01 | 0.0000 | 2127.0 | 9.64% | 2317.7 | 8.76% | motif file (matrix) | svg |
| 83 | T C G A T G C A A C T G G T C A A G T C A G T C A G T C A G T C A G C T T G A C | LRF(Zf)/Erythroblasts-ZBTB7A-ChIP-Seq(GSE74977)/Homer | 1e-5 | -1.271e+01 | 0.0000 | 9620.0 | 43.58% | 11132.7 | 42.07% | motif file (matrix) | svg |
| 84 | C T G A T G C A T A G C T G A C T A C G T C A G C T G A G C T A T C A G G A C T | ELF1(ETS)/Jurkat-ELF1-ChIP-Seq(SRA014231)/Homer | 1e-5 | -1.264e+01 | 0.0000 | 2572.0 | 11.65% | 2831.4 | 10.70% | motif file (matrix) | svg |
| 85 | C G T A C T A G C A T G A G C T A C T G C G A T A T C G C G T A G T C A G T C A | Tbet(T-box)/CD8-Tbet-ChIP-Seq(GSE33802)/Homer | 1e-5 | -1.257e+01 | 0.0000 | 1968.0 | 8.92% | 2137.9 | 8.08% | motif file (matrix) | svg |
| 86 | C T G A T C G A C G T A A T G C C G T A C G T A C G A T C T A G T C A G G A T C | Sox15(HMG)/CPA-Sox15-ChIP-Seq(GSE62909)/Homer | 1e-5 | -1.242e+01 | 0.0000 | 2201.0 | 9.97% | 2406.7 | 9.10% | motif file (matrix) | svg |
| 87 | C T A G A C T G T G C A A G T C C G T A A C T G A C T G A C G T C T A G C G A T T A C G A G T C | ZEB2(Zf)/SNU398-ZEB2-ChIP-Seq(GSE103048)/Homer | 1e-5 | -1.236e+01 | 0.0000 | 3947.0 | 17.88% | 4432.5 | 16.75% | motif file (matrix) | svg |
| 88 | C T G A A G T C C G A T A G C T A T G C G T A C A C G T A T C G C A G T G C A T | Elf4(ETS)/BMDM-Elf4-ChIP-Seq(GSE88699)/Homer | 1e-5 | -1.216e+01 | 0.0000 | 3369.0 | 15.26% | 3761.5 | 14.22% | motif file (matrix) | svg |
| 89 | A G T C C G A T C T G A C G T A A C G T C A G T T C A G T G A C | Isl1(Homeobox)/Neuron-Isl1-ChIP-Seq(GSE31456)/Homer | 1e-5 | -1.198e+01 | 0.0000 | 3675.0 | 16.65% | 4120.6 | 15.57% | motif file (matrix) | svg |
| 90 | T C A G A G C T A C G T A C G T G T A C G A T C C G T A C T A G C A T G G T C A C G T A T C G A | STAT4(Stat)/CD4-Stat4-ChIP-Seq(GSE22104)/Homer | 1e-5 | -1.160e+01 | 0.0000 | 2194.0 | 9.94% | 2407.5 | 9.10% | motif file (matrix) | svg |
| 91 | C G T A T A C G T C G A A C T G A C T G C G T A C G T A T A C G A G C T T A C G | PU.1(ETS)/ThioMac-PU.1-ChIP-Seq(GSE21512)/Homer | 1e-4 | -1.148e+01 | 0.0001 | 1272.0 | 5.76% | 1354.2 | 5.12% | motif file (matrix) | svg |
| 92 | T C A G G C T A T C A G C A G T T G A C G T C A A G T C A T C G T G C A G T A C C A G T G A T C | Npas4(bHLH)/Neuron-Npas4-ChIP-Seq(GSE127793)/Homer | 1e-4 | -1.147e+01 | 0.0001 | 2894.0 | 13.11% | 3218.9 | 12.17% | motif file (matrix) | svg |
| 93 | A T G C G A C T A G C T C T A G C G T A C T A G C G A T C T A G A T C G G A T C | Nkx2.2(Homeobox)/NPC-Nkx2.2-ChIP-Seq(GSE61673)/Homer | 1e-4 | -1.139e+01 | 0.0001 | 4869.0 | 22.06% | 5527.7 | 20.89% | motif file (matrix) | svg |
| 94 | T G A C T A G C T C A G T C G A T C G A C G T A A G T C C G T A C G T A C G A T C T A G T A C G | Sox7(HMG)/ESC-Sox7-ChIP-Seq(GSE133899)/Homer | 1e-4 | -1.131e+01 | 0.0001 | 627.0 | 2.84% | 633.5 | 2.39% | motif file (matrix) | svg |
| 95 | T G A C C T A G A C T G T A G C C G A T A C T G A T G C C A T G A T C G A T C G A T C G T A G C C T G A T A G C G C T A A C T G C G T A A G C T C G T A C T G A | GATA:SCL(Zf,bHLH)/Ter119-SCL-ChIP-Seq(GSE18720)/Homer | 1e-4 | -1.125e+01 | 0.0001 | 228.0 | 1.03% | 204.4 | 0.77% | motif file (matrix) | svg |
| 96 | C G T A C T G A C G T A C T A G T C G A C T A G A C T G C G T A C G T A T A C G A G C T A T C G | SpiB(ETS)/OCILY3-SPIB-ChIP-Seq(GSE56857)/Homer | 1e-4 | -1.121e+01 | 0.0001 | 591.0 | 2.68% | 594.9 | 2.25% | motif file (matrix) | svg |
| 97 | A T C G T C G A G A C T A T C G T G A C A C G T C T A G A C T G C G T A A C T G A G T C G T A C | ZNF415(Zf)/HEK293-ZNF415.GFP-ChIP-Seq(GSE58341)/Homer | 1e-4 | -1.115e+01 | 0.0001 | 2271.0 | 10.29% | 2501.5 | 9.45% | motif file (matrix) | svg |
| 98 | C G T A T G A C T A G C T G C A A C T G A C T G C G T A C G T A T C A G G A C T | ELF3(ETS)/PDAC-ELF3-ChIP-Seq(GSE64557)/Homer | 1e-4 | -1.095e+01 | 0.0001 | 1940.0 | 8.79% | 2122.1 | 8.02% | motif file (matrix) | svg |
| 99 | T A G C C G A T A C G T A G C T A G C T A G T C A T G C A G T C A C T G A T G C A T G C G C T A | E2F7(E2F)/Hela-E2F7-ChIP-Seq(GSE32673)/Homer | 1e-4 | -1.085e+01 | 0.0001 | 960.0 | 4.35% | 1007.4 | 3.81% | motif file (matrix) | svg |
| 100 | A T G C T A C G A G C T T G C A C G T A C G A T A C G T C T G A | DLX5(Homeobox)/BasalGanglia-Dlx5-ChIP-seq(GSE124936)/Homer | 1e-4 | -1.082e+01 | 0.0001 | 1318.0 | 5.97% | 1412.1 | 5.34% | motif file (matrix) | svg |
| 101 | C G T A G C T A C G A T C T A G A C G T G T C A C G T A C G T A A G T C C G T A T G C A T A C G | FoxL2(Forkhead)/Ovary-FoxL2-ChIP-Seq(GSE60858)/Homer | 1e-4 | -1.065e+01 | 0.0001 | 1493.0 | 6.76% | 1613.7 | 6.10% | motif file (matrix) | svg |
| 102 | C A G T A C T G C G T A G T C A C G T A A G C T G A T C A G C T A C T G C G T A A G T C G T C A A G T C A G T C A T C G | Six4(Homeobox)/MCF7-SIX4-ChIP-Seq(Encode)/Homer | 1e-4 | -1.051e+01 | 0.0001 | 116.0 | 0.53% | 93.2 | 0.35% | motif file (matrix) | svg |
| 103 | T G C A T C G A T A G C G T A C T C A G C T A G G T C A G C T A T C A G G A C T | ETS(ETS)/Promoter/Homer | 1e-4 | -1.010e+01 | 0.0002 | 1399.0 | 6.34% | 1511.7 | 5.71% | motif file (matrix) | svg |
| 104 | C T G A A G C T A C G T A C G T A G T C G A C T G A C T C T G A C T G A C T A G C G T A C G T A | STAT6(Stat)/CD4-Stat6-ChIP-Seq(GSE22104)/Homer | 1e-4 | -1.000e+01 | 0.0002 | 1138.0 | 5.16% | 1215.3 | 4.59% | motif file (matrix) | svg |
| 105 | T G C A G T A C C G T A A T C G A C T G A C G T C T A G C G A T T C G A A G T C | ZEB1(Zf)/PDAC-ZEB1-ChIP-Seq(GSE64557)/Homer | 1e-4 | -9.806e+00 | 0.0002 | 6324.0 | 28.65% | 7271.4 | 27.48% | motif file (matrix) | svg |
| 106 | A G T C G A C T A C T G G A T C G T A C C G T A T G A C A G T C C G A T A G C T A C G T A C G T C T A G G A C T C T G A | ZNF7(Zf)/HepG2-ZNF7.Flag-ChIP-Seq(Encode)/Homer | 1e-4 | -9.790e+00 | 0.0002 | 1049.0 | 4.75% | 1116.2 | 4.22% | motif file (matrix) | svg |
| 107 | C T A G C T G A T C A G A T C G A G C T C A T G G A C T A G T C C T G A T G C A | Tbx6(T-box)/ESC-Tbx6-ChIP-Seq(GSE93524)/Homer | 1e-4 | -9.682e+00 | 0.0003 | 2567.0 | 11.63% | 2862.8 | 10.82% | motif file (matrix) | svg |
| 108 | A G T C A G T C C G A T A C G T A C G T A C T G A C G T A G C T A G T C A G T C | Sox4(HMG)/proB-Sox4-ChIP-Seq(GSE50066)/Homer | 1e-4 | -9.571e+00 | 0.0003 | 1916.0 | 8.68% | 2111.0 | 7.98% | motif file (matrix) | svg |
| 109 | C G T A C G T A C G T A G C A T G C A T A C T G G T A C G A C T C T A G G C T A T A C G G A C T T G A C C G T A A G C T | NFE2L2(bZIP)/HepG2-NFE2L2-ChIP-Seq(Encode)/Homer | 1e-3 | -8.878e+00 | 0.0006 | 119.0 | 0.54% | 100.3 | 0.38% | motif file (matrix) | svg |
| 110 | C A G T A C T G T C A G T G C A G C T A A T G C T C G A A T C G G T C A T G C A | ZNF189(Zf)/HEK293-ZNF189.GFP-ChIP-Seq(GSE58341)/Homer | 1e-3 | -8.559e+00 | 0.0008 | 2348.0 | 10.64% | 2623.9 | 9.92% | motif file (matrix) | svg |
| 111 | A T G C A G T C C T G A A G T C C G A T A C G T A G T C A G T C A C G T A T C G G A C T A C G T | Etv2(ETS)/ES-ER71-ChIP-Seq(GSE59402)/Homer | 1e-3 | -8.509e+00 | 0.0008 | 2843.0 | 12.88% | 3200.8 | 12.10% | motif file (matrix) | svg |
| 112 | T A C G C T G A T C G A C G A T C T A G C T A G T C G A C T G A T C G A T C G A C G T A T C G A G C A T C A T G C G T A T A C G G C A T T G A C C G T A A G C T | NFAT:AP1(RHD,bZIP)/Jurkat-NFATC1-ChIP-Seq(Jolma\_et\_al.)/Homer | 1e-3 | -8.419e+00 | 0.0009 | 342.0 | 1.55% | 337.9 | 1.28% | motif file (matrix) | svg |
| 113 | A C G T C T A G C G T A A G T C G T A C A C G T A C G T A C G T G T C A G T A C T G A C G A C T | Nur77(NR)/K562-NR4A1-ChIP-Seq(GSE31363)/Homer | 1e-3 | -8.291e+00 | 0.0010 | 357.0 | 1.62% | 354.2 | 1.34% | motif file (matrix) | svg |
| 114 | T G C A C T G A A G T C G T C A A C T G A C T G C G T A C G T A C T G A A G C T | EWS:FLI1-fusion(ETS)/SK\_N\_MC-EWS:FLI1-ChIP-Seq(SRA014231)/Homer | 1e-3 | -8.282e+00 | 0.0010 | 1834.0 | 8.31% | 2031.4 | 7.68% | motif file (matrix) | svg |
| 115 | T G C A C T G A A T G C G T C A A C T G A C T G C G T A C G T A C T A G A G C T | Ets1-distal(ETS)/CD4+-PolII-ChIP-Seq(Barski\_et\_al.)/Homer | 1e-3 | -8.268e+00 | 0.0010 | 702.0 | 3.18% | 737.8 | 2.79% | motif file (matrix) | svg |
| 116 | T A C G T C G A C A G T A C T G G C T A A T G C C G A T G T A C C G T A A C T G T A G C C G T A | NF-E2(bZIP)/K562-NFE2-ChIP-Seq(GSE31477)/Homer | 1e-3 | -8.235e+00 | 0.0010 | 135.0 | 0.61% | 118.1 | 0.45% | motif file (matrix) | svg |
| 117 | T A C G T A C G G T A C A T C G A C T G T A C G T C G A C T G A T C G A A T C G | E2F6(E2F)/Hela-E2F6-ChIP-Seq(GSE31477)/Homer | 1e-3 | -8.194e+00 | 0.0010 | 4407.0 | 19.96% | 5039.3 | 19.04% | motif file (matrix) | svg |
| 118 | T C A G C T A G C T A G T G C A C G A T C G A T C G T A C T A G | GSC(Homeobox)/FrogEmbryos-GSC-ChIP-Seq(DRA000576)/Homer | 1e-3 | -7.905e+00 | 0.0014 | 2143.0 | 9.71% | 2394.1 | 9.05% | motif file (matrix) | svg |
| 119 | G T C A G C A T A C T G G T A C G A C T A C T G G C T A A T C G C A G T G T A C C G T A A G C T | Nrf2(bZIP)/Lymphoblast-Nrf2-ChIP-Seq(GSE37589)/Homer | 1e-3 | -7.790e+00 | 0.0015 | 118.0 | 0.53% | 102.1 | 0.39% | motif file (matrix) | svg |
| 120 | G C A T G C A T C T G A A C G T C T G A A C G T C G T A C G T A C G T A A G T C G T C A G T C A | Foxf1(Forkhead)/Lung-Foxf1-ChIP-Seq(GSE77951)/Homer | 1e-3 | -7.535e+00 | 0.0020 | 1658.0 | 7.51% | 1837.7 | 6.95% | motif file (matrix) | svg |
| 121 | A G T C G A T C A G T C C G T A A T C G C A G T A G T C G T A C C T G A A C T G T C A G A G C T A G C T A G C T A G C T | PRDM15(Zf)/ESC-Prdm15-ChIP-Seq(GSE73694)/Homer | 1e-3 | -7.164e+00 | 0.0028 | 3341.0 | 15.14% | 3805.1 | 14.38% | motif file (matrix) | svg |
| 122 | A G T C A T C G C T A G A G C T G A C T C T A G A G T C A G T C G C T A C A G T T C A G T C A G G A T C C T G A T C G A G A T C | RFX(HTH)/K562-RFX3-ChIP-Seq(SRA012198)/Homer | 1e-3 | -7.127e+00 | 0.0029 | 300.0 | 1.36% | 298.1 | 1.13% | motif file (matrix) | svg |
| 123 | G A T C G C T A C A G T A C G T T A C G A G T C A T G C C T A G A G T C T C G A | Zfp57(Zf)/H1-ZFP57.HA-ChIP-Seq(GSE115387)/Homer | 1e-3 | -7.000e+00 | 0.0033 | 3110.0 | 14.09% | 3537.7 | 13.37% | motif file (matrix) | svg |
| 124 | C T G A A C G T A C G T A C G T A G T C G A C T C G A T C T G A A C T G C G T A C G T A T C G A | STAT5(Stat)/mCD4+-Stat5-ChIP-Seq(GSE12346)/Homer | 1e-3 | -6.922e+00 | 0.0035 | 770.0 | 3.49% | 825.6 | 3.12% | motif file (matrix) | svg |
| 125 | T G C A T A G C G A C T T G C A T G A C T G C A C G T A A G C T A G C T A G T C A G T C G T A C | GFY(?)/Promoter/Homer | 1e-2 | -6.414e+00 | 0.0058 | 154.0 | 0.70% | 144.3 | 0.55% | motif file (matrix) | svg |
| 126 | C G A T C T A G A C G T A C G T A C G T C G T A A G C T C G A T A G C T C G T A C T A G T A G C | FoxD3(forkhead)/ZebrafishEmbryo-Foxd3.biotin-ChIP-seq(GSE106676)/Homer | 1e-2 | -6.389e+00 | 0.0059 | 1750.0 | 7.93% | 1959.8 | 7.41% | motif file (matrix) | svg |
| 127 | C T G A T C A G G C T A A G C T A G T C G A C T C T G A C T A G T G C A C T G A A G T C G T A C G A T C A C T G T C G A | ZBTB12(Zf)/HEK293-ZBTB12.GFP-ChIP-Seq(GSE58341)/Homer | 1e-2 | -6.206e+00 | 0.0070 | 1175.0 | 5.32% | 1296.2 | 4.90% | motif file (matrix) | svg |
| 128 | T C G A C A G T A C T G A G C T C G T A C G T A A C G T A C G T C T G A T A G C | Dlx3(Homeobox)/Kerainocytes-Dlx3-ChIP-Seq(GSE89884)/Homer | 1e-2 | -6.202e+00 | 0.0070 | 1082.0 | 4.90% | 1189.9 | 4.50% | motif file (matrix) | svg |
| 129 | T C G A C G T A A G T C A G C T C G T A A G T C T C G A G C T A G A C T C G A T A G T C A G T C A G T C C T G A T C A G T G C A T C G A C A G T A T C G A G T C | GFY-Staf(?,Zf)/Promoter/Homer | 1e-2 | -5.985e+00 | 0.0086 | 124.0 | 0.56% | 114.9 | 0.43% | motif file (matrix) | svg |
| 130 | C T A G A G C T G A C T C A T G A G T C A G T C G T C A C A G T C T A G T C A G G T A C C T G A T C G A G A T C T G A C | Rfx2(HTH)/LoVo-RFX2-ChIP-Seq(GSE49402)/Homer | 1e-2 | -5.371e+00 | 0.0157 | 325.0 | 1.47% | 336.1 | 1.27% | motif file (matrix) | svg |
| 131 | T G A C T C G A C T G A C T G A A T G C G A T C C T A G T A C G G A C T G A C T G A T C T C G A C T G A C T G A A T G C G A T C C T A G A T C G G A C T G A C T | Tcfcp2l1(CP2)/mES-Tcfcp2l1-ChIP-Seq(GSE11431)/Homer | 1e-2 | -5.271e+00 | 0.0173 | 419.0 | 1.90% | 443.0 | 1.67% | motif file (matrix) | svg |
| 132 | C T G A C T A G A C T G G C A T C T A G G C A T A T C G C T G A C G T A G T C A | Tbx21(T-box)/GM12878-TBX21-ChIP-Seq(Encode)/Homer | 1e-2 | -5.271e+00 | 0.0173 | 1888.0 | 8.55% | 2137.5 | 8.08% | motif file (matrix) | svg |
| 133 | T G C A C T A G C T A G C T G A C A T G A C T G T G C A G A T C G T C A T G C A G T C A G T C A A G C T C T A G G C A T | ZNF675(Zf)/HEK293-ZNF675.GFP-ChIP-Seq(GSE58341)/Homer | 1e-2 | -5.125e+00 | 0.0197 | 284.0 | 1.29% | 292.4 | 1.11% | motif file (matrix) | svg |
| 134 | A T C G G A C T C G A T C G T A C G T A C A G T G A T C G A T C G A T C A G C T | Otx2(Homeobox)/EpiLC-Otx2-ChIP-Seq(GSE56098)/Homer | 1e-2 | -5.046e+00 | 0.0211 | 1523.0 | 6.90% | 1715.5 | 6.48% | motif file (matrix) | svg |
| 135 | G A C T C A G T G A T C G A T C A C G T G A T C C T G A T A C G C G T A G T C A | STAT6(Stat)/Macrophage-Stat6-ChIP-Seq(GSE38377)/Homer | 1e-2 | -4.950e+00 | 0.0231 | 1143.0 | 5.18% | 1275.4 | 4.82% | motif file (matrix) | svg |
| 136 | T C A G G A C T C A G T C T G A A G C T C T A G G A C T T G C A C T G A A G T C | HLF(bZIP)/HSC-HLF.Flag-ChIP-Seq(GSE69817)/Homer | 1e-2 | -4.712e+00 | 0.0291 | 1216.0 | 5.51% | 1363.7 | 5.15% | motif file (matrix) | svg |
| 137 | C G A T T A C G T G C A G T A C G A T C G A C T A G C T A C G T A T C G G T A C G A T C G T A C G A T C G T C A | PPARE(NR),DR1/3T3L1-Pparg-ChIP-Seq(GSE13511)/Homer | 1e-2 | -4.620e+00 | 0.0317 | 3046.0 | 13.80% | 3509.4 | 13.26% | motif file (matrix) | svg |
